# Supplementary material for: Genetic integration of behavioural and endocrine components of the stress response
Source: eLife. 2022 Feb 11;11:e67126. doi: 10.7554/eLife.67126 (PMC8837200; doi:10.7554/eLife.67126)
Supplement: Supplementary file 1. [file elife-67126-supp1.docx]

Fixed effects estimates from the full multivariate animal model.

| **Trait** | **Parameter** | **Estimate** | **SE** | **z** |
| --- | --- | --- | --- | --- |
| Relative area | (Intercept) | -0.198 | 0.153 | -1.291 |
|  | Age | 0.04 | 0.04 | 1.003 |
|  | Generation (1) | -0.118 | 0.107 | -1.103 |
|  | Generation (2) | 0.268 | 0.115 | 2.327 |
|  | Generation (3) | 0.657 | 0.282 | 2.331 |
|  | Generation (S F1) | 0.868 | 0.274 | 3.166 |
|  | Order | -0.004 | 0.004 | -1.067 |
|  | Repeat | -0.07 | 0.015 | -4.584 |
|  | Sex (Male) | 0.142 | 0.039 | 3.614 |
|  | Temperature | -0.026 | 0.023 | -1.161 |
|  | Time of day | 0.18 | 0.122 | 1.47 |
| Time in the middle | (Intercept) | -0.344 | 0.156 | -2.209 |
|  | Age | 0.084 | 0.041 | 2.047 |
|  | Generation (1) | 0.064 | 0.11 | 0.578 |
|  | Generation (2) | 0.359 | 0.12 | 3 |
|  | Generation (3) | 0.582 | 0.287 | 2.028 |
|  | Generation (S F1) | 1.077 | 0.279 | 3.865 |
|  | Order | -0.004 | 0.004 | -0.943 |
|  | Repeat | -0.098 | 0.015 | -6.344 |
|  | Sex (Male) | 0.346 | 0.04 | 8.721 |
|  | Temperature | 0.025 | 0.023 | 1.109 |
|  | Time of day | 0.271 | 0.124 | 2.184 |
| Track length | (Intercept) | -0.694 | 0.143 | -4.861 |
|  | Age | -0.167 | 0.038 | -4.436 |
|  | Generation (1) | 0.567 | 0.097 | 5.871 |
|  | Generation (2) | 0.469 | 0.106 | 4.41 |
|  | Generation (3) | 0.703 | 0.266 | 2.646 |
|  | Generation (S F1) | 0.233 | 0.257 | 0.907 |
|  | Order | -0.009 | 0.004 | -2.419 |
|  | Repeat | 0.147 | 0.015 | 10.05 |
|  | Sex (Male) | -0.155 | 0.04 | -3.862 |
|  | Temperature | 0.04 | 0.021 | 1.894 |
|  | Time of day | 0.028 | 0.115 | 0.247 |
| √Freezings | (Intercept) | 0.431 | 0.158 | 2.731 |
|  | Age | 0.157 | 0.041 | 3.862 |
|  | Generation (1) | -0.236 | 0.105 | -2.236 |
|  | Generation (2) | -0.252 | 0.116 | -2.163 |
|  | Generation (3) | -0.193 | 0.293 | -0.66 |
|  | Generation (S F1) | 0.088 | 0.284 | 0.311 |
|  | Order | -0.002 | 0.004 | -0.518 |
|  | Repeat | -0.138 | 0.016 | -8.45 |
|  | Sex (Male) | -0.022 | 0.04 | -0.535 |
|  | Temperature | 0.037 | 0.023 | 1.616 |
|  | Time of day | 0.058 | 0.127 | 0.456 |
| -ln Emergence time | (Intercept) | 0.087 | 0.334 | 0.26 |
|  | Age | 0.002 | 0.045 | 0.046 |
|  | Generation (3) | -0.713 | 0.668 | -1.068 |
|  | Generation (S F1) | -0.593 | 0.668 | -0.889 |
|  | Order | -0.004 | 0.005 | -0.737 |
|  | Repeat | 0.073 | 0.045 | 1.597 |
|  | Sex (Male) | 0.259 | 0.052 | 4.988 |
|  | Temperature | 0.08 | 0.039 | 2.048 |
|  | Time of day | -0.265 | 0.326 | -0.811 |
| ln Cortisol | (Intercept) | 1.054 | 0.334 | 3.154 |
|  | Age | 0 | 0.029 | 0.007 |
|  | Order | -0.001 | 0.004 | -0.318 |
|  | Sex (Male) | -0.846 | 0.079 | -10.668 |
|  | Stressor exposure (first) | 0.672 | 0.035 | 19.316 |
|  | Temperature | -0.1 | 0.048 | -2.1 |
|  | Time of day | -1.337 | 0.465 | -2.876 |
|  | Weight | 0.261 | 0.055 | 4.74 |
|  | Weight x Sex (Male) | -0.158 | 0.102 | -1.554 |
