# Supplementary material for: Genetic integration of behavioural and endocrine components of the stress response
Source: eLife. 2022 Feb 11;11:e67126. doi: 10.7554/eLife.67126 (PMC8837200; doi:10.7554/eLife.67126)
Supplement: Supplementary file 3. [file elife-67126-supp3.docx]

Group (co)variance matrix from the full multivariate animal model.

|  | Relative area | Time in the middle | Track length | √Freezings | -ln Emergence time | ln Cortisol |
| --- | --- | --- | --- | --- | --- | --- |
| Relative area | 0.05 ± 0.01 |  |  |  |  |  |
| Time in the middle | 0.03 ± 0.01 | 0.06 ± 0.02 |  |  |  |  |
| Track length | -0.02 ± 0.01 | -0.03 ± 0.01 | 0.03 ± 0.01 |  |  |  |
| √Freezings | 0.01 ± 0.01 | 0.03 ± 0.01 | -0.02 ± 0.01 | 0.05 ± 0.01 |  |  |
| -ln Emergence time | -0.00 ± 0.01 | -0.02 ± 0.01 | 0.01 ± 0.01 | -0.03 ± 0.01 | 0.03 ± 0.01 |  |
| ln Cortisol | -0.01 ± 0.01 | 0.02 ± 0.01 | -0.03 ± 0.01 | 0.02 ± 0.01 | 0.01 ± 0.01 | 0.07 ± 0.03 |
