# Supplementary material for: Genetic integration of behavioural and endocrine components of the stress response
Source: eLife. 2022 Feb 11;11:e67126. doi: 10.7554/eLife.67126 (PMC8837200; doi:10.7554/eLife.67126)
Supplement: Supplementary file 4. [file elife-67126-supp4.docx]

Residual variance-correlation matrix from the full multivariate animal model.

|  | Relative area | Time in the middle | Track length | √Freezings | -ln Emergence time | ln Cortisol |
| --- | --- | --- | --- | --- | --- | --- |
| Relative area | 0.55 ± 0.02 | 0.65 ± 0.01 | -0.64 ± 0.01 | 0.41 ± 0.02 | - | - |
| Time in the middle |  | 0.56 ± 0.02 | -0.59 ± 0.01 | 0.51 ± 0.02 | - | - |
| Track length |  |  | 0.50 ± 0.02 | -0.68 ± 0.01 | - | - |
| √Freezings |  |  |  | 0.63 ± 0.02 |  |  |
| -ln Emergence time |  |  |  |  | 0.79 ± 0.04 | - |
| ln Cortisol |  |  |  |  |  | 0.35 ± 0.02 |
