# Supplementary material for: Genetic integration of behavioural and endocrine components of the stress response
Source: eLife. 2022 Feb 11;11:e67126. doi: 10.7554/eLife.67126 (PMC8837200; doi:10.7554/eLife.67126)
Supplement: Supplementary file 5. [file elife-67126-supp5.docx]

Among-individual (co)variance matrix from the multivariate model excluding genetic effects.

|  | Relative area | Time in the middle | Track length | √Freezings | -ln Emergence time | Shoaling tendency | ln Cortisol |
| --- | --- | --- | --- | --- | --- | --- | --- |
| Relative area | 0.25 (0.211,0.288) | 0.747 (0.692,0.801) | -0.728 (-0.78,-0.674) | 0.49 (0.392,0.576) | 0.032 (-0.219,0.273) | 0.198 (-0.054,0.436) | -0.002 (-0.186,0.16) |
| Time in the middle | 0.19 (0.154,0.223) | 0.258 (0.216,0.298) | -0.645 (-0.702,-0.577) | 0.601 (0.522,0.68) | -0.134 (-0.373,0.113) | 0.025 (-0.22,0.287) | 0.123 (-0.05,0.299) |
| Track length | -0.203 (-0.237,-0.167) | -0.182 (-0.218,-0.148) | 0.31 (0.265,0.35) | -0.76 (-0.808,-0.709) | 0.168 (-0.046,0.399) | -0.053 (-0.286,0.168) | -0.003 (-0.159,0.156) |
| √Freezings | 0.123 (0.09,0.155) | 0.153 (0.118,0.187) | -0.212 (-0.248,-0.173) | 0.251 (0.21,0.297) | -0.333 (-0.605,-0.101) | -0.066 (-0.307,0.185) | -0.038 (-0.231,0.131) |
| -ln Emergence time | 0.006 (-0.038,0.044) | -0.024 (-0.066,0.017) | 0.033 (-0.01,0.073) | -0.059 (-0.101,-0.02) | 0.127 (0.06,0.193) | 0.319 (0.001,0.666) | -0.236 (-0.502,0.015) |
| Shoaling tendency | 0.043 (-0.009,0.093) | 0.006 (-0.048,0.058) | -0.013 (-0.067,0.036) | -0.014 (-0.066,0.036) | 0.049 (0.004,0.098) | 0.185 (0.102,0.267) | -0.01 (-0.243,0.237) |
| ln Cortisol | 0 (-0.038,0.033) | 0.026 (-0.011,0.063) | -0.001 (-0.037,0.035) | -0.008 (-0.046,0.028) | -0.035 (-0.069,0.002) | -0.002 (-0.045,0.037) | 0.176 (0.133,0.222) |

Shoaling tendency is included as there is significant among-individual variance in this trait. Among-individual variances provided on the shaded diagonal, with among-individual covariances below and among-individual correlations above. 95% confidence intervals in parentheses are estimated from 5000 bootstrapped replicates.
