# Supplementary material for: Genetic integration of behavioural and endocrine components of the stress response
Source: eLife. 2022 Feb 11;11:e67126. doi: 10.7554/eLife.67126 (PMC8837200; doi:10.7554/eLife.67126)
Supplement: Supplementary file 6. [file elife-67126-supp6.docx]

Eigen decomposition of the **G** matrix.

|  | **Eigen vectors** | | | | | |
| --- | --- | --- | --- | --- | --- | --- |
|  | 1 | 2 | 3 | 4 | 5 | 6 |
| Eigen value | 0.47 | 0.16 | 0.11 | 0.04 | 0.01 | 0.01 |
| Percentage of total | 59.5% | 20.2% | 14.1% | 4.5% | 1.0% | 0.7% |
|  | Trait loadings | | | | | |
| *Relative area covered* | -0.33 | -0.53 | -0.33 | 0.32 | -0.24 | 0.58 |
| *Time in the middle* | -0.45 | -0.50 | 0.10 | -0.41 | 0.57 | -0.20 |
| *Track length* | 0.52 | -0.17 | 0.26 | 0.40 | 0.61 | 0.32 |
| √*Freezings* | -0.50 | 0.62 | 0.17 | -0.03 | 0.28 | 0.51 |
| -ln *Emergence time* | 0.21 | -0.20 | 0.58 | -0.54 | 0.35 | 0.42 |
| ln *Cortisol* | 0.35 | 0.11 | -0.67 | -0.53 | 0.21 | 0.30 |
