# Supplementary material for: Genetic integration of behavioural and endocrine components of the stress response
Source: eLife. 2022 Feb 11;11:e67126. doi: 10.7554/eLife.67126 (PMC8837200; doi:10.7554/eLife.67126)
Supplement: Transparent reporting form [file elife-67126-transrepform1.docx]

***eLife’s* transparent reporting form**

We encourage authors to provide detailed information *within their submission* to facilitate the interpretation and replication of experiments. Authors can upload supporting documentation to indicate the use of appropriate reporting guidelines for health-related research (see [EQUATOR Network](http://www.equator-network.org/%20)), life science research (see the [BioSharing Information Resource](https://biosharing.org/)), or the [ARRIVE guidelines](http://www.plosbiology.org/article/info:doi/10.1371/journal.pbio.1000412) for reporting work involving animal research. Where applicable, authors should refer to any relevant reporting standards documents in this form.

If you have any questions, please consult our Journal Policies and/or contact us: [editorial@elifesciences.org](mailto:editorial@elifesciences.org).

**Sample-size estimation**

- You should state whether an appropriate sample size was computed when the study was being designed
- You should state the statistical method of sample size computation and any required assumptions
- If no explicit power analysis was used, you should describe how you decided what sample (replicate) size (number) to use

Please outline where this information can be found within the submission (e.g., sections or figure legends), or explain why this information doesn’t apply to your submission:

Our study comprises behavioural and endocrine samples from a pedigreed captive population created in a half-sib breeding design (where each male is mated to several females). A subset of the data has previously been published, demonstrating genetic variation in (and covariation among) behavioural traits (White & Wilson 2018 Heredity; White, Houslay & Wilson 2018 Heredity). We built upon this data through additional breeding to generate a sample size large enough to feasibly demonstrate the existence of genetic variance in waterborne Cortisol release (having already shown individual variation in Cortisol in a small sample; Houslay et al 2019 Gen. Comp. Endocrinol.). Given issues with breeding in the past, but with constraints of number of breeding tanks, we aimed for a minimum of 80 breeding females (with 1 male per 3 females, and with repeated litters from females as possible), with the aim of adding a minimum of 500 individuals to the pedigree with both endocrine and behavioural measures. Where possible, we also used fish from previous generations within the pedigree so as to generate more power via the pedigree structure.

**Replicates**

- You should report how often each experiment was performed
- You should include a definition of biological versus technical replication
- The data obtained should be provided and sufficient information should be provided to indicate the number of independent biological and/or technical replicates
- If you encountered any outliers, you should describe how these were handled
- Criteria for exclusion/inclusion of data should be clearly stated
- High-throughput sequence data should be uploaded before submission, with a private link for reviewers provided (these are available from both GEO and ArrayExpress)

Please outline where this information can be found within the submission (e.g., sections or figure legends), or explain why this information doesn’t apply to your submission:

We report sample size and replicates in the Results section. Briefly, we obtained (multivariate) behavioural data from 5,966 trials (3,379 Open Field Trials, 1,548 Emergence Trials and 1,039 Shoaling Trials) on 1,384 individual fish. The number of individuals phenotyped (OFTs = 1,365, ETs = 806, STs = 532) and the mean number of observations per fish (OFTs = 2.5, ETs = 1.9, STs = 2.0) varied across the behavioural data types. We also obtained 1,238 waterborne assays of cortisol levels for 629 fish (almost all from the final generation). The handling and confinement stressor applied for this assay was performed 3 times (at 48h intervals) for all fish tested, but the holding water sample was only processed for GC content at two time points (the first and last confinement).

Further information on behavioural phenotyping is provided in Materials & Methods: Overview of behavioural phenotyping. Briefly, the first offspring generation experienced 4 repeat open field trials (OFTs) over a 2-week period, with at least 48h between trials. Subsequent generations experienced 4 repeat behavioural trials, alternating 2 OFTs with 2 emergence trials (ETs). For the final 2 generations, we extended the OFTs by including a shoaling trial (ST) at the end of each OFT.

Further information on waterborne hormone sampling is provided in Materials & Methods: Waterborne hormone sampling. Briefly, individuals were left undisturbed in their home tanks for a minimum of two weeks following behavioural phenotyping. Waterborne hormone sampling was then conducted over a 5-day period that included three handling and confinement stressor exposures with 48h between each.

**Statistical reporting**

- Statistical analysis methods should be described and justified
- Raw data should be presented in figures whenever informative to do so (typically when N per group is less than 10)
- For each experiment, you should identify the statistical tests used, exact values of N, definitions of center, methods of multiple test correction, and dispersion and precision measures (e.g., mean, median, SD, SEM, confidence intervals; and, for the major substantive results, a measure of effect size (e.g., Pearson's r, Cohen's d)
- Report exact p-values wherever possible alongside the summary statistics and 95% confidence intervals. These should be reported for all key questions and not only when the p-value is less than 0.05.

Please outline where this information can be found within the submission (e.g., sections or figure legends), or explain why this information doesn’t apply to your submission:

Statistical analysis methods are described in detail in Materials & Methods: Statistical methods. These involve maximum likelihood estimation of quantitative genetic parameters, with significance determined using likelihood ratio tests. We use a parametric bootstrapping approach to estimate 95% confidence intervals for the genetic (co)variance matrix as well as for our eigen analysis and for visualization of results.

(For large datasets, or papers with a very large number of statistical tests, you may upload a single table file with tests, Ns, etc., with reference to sections in the manuscript.)

**Group allocation**

- Indicate how samples were allocated into experimental groups (in the case of clinical studies, please specify allocation to treatment method); if randomization was used, please also state if restricted randomization was applied
- Indicate if masking was used during group allocation, data collection and/or data analysis

Please outline where this information can be found within the submission (e.g., sections or figure legends), or explain why this information doesn’t apply to your submission:

Our study does not include an experimental manipulation. Individuals were assigned to mixed tanks (16-20 adults, with an even mix of males and females) haphazardly, with groups necessarily established sequentially as sufficient individuals from multiple families reached a large enough size that we deemed the procedure to be safe. More details are provided in Materials & Methods: Husbandry and breeding.

**Additional data files (“source data”)**

- We encourage you to upload relevant additional data files, such as numerical data that are represented as a graph in a figure, or as a summary table
- Where provided, these should be in the most useful format, and they can be uploaded as “Source data” files linked to a main figure or table
- Include model definition files including the full list of parameters used
- Include code used for data analysis (e.g., R, MatLab)
- Avoid stating that data files are “available upon request”

Please indicate the figures or tables for which source data files have been provided:

None of the figures or tables are data summaries, but rather represent output fromo statistical models. We have uploaded the code used for data analysis and generating tables and figures, along with the full data set, to Dryad.
